# Editorial: Parents With Mental and/or Substance Use Disorders and Their Children

**DOI:** 10.3389/fpsyt.2019.00915

**Published:** 2019-12-10

**Authors:** Joanne Nicholson, Giovanni de Girolamo, Beate Schrank

**Affiliations:** ^1^Institute for Behavioral Health, Brandeis University, Waltham, MA, United States; ^2^IRCCS Centro San Giovanni di Dio Fatebenefratelli, Brescia, Italy; ^3^Department of Psychiatry and Psychotherapy, Karl Landsteiner University of Health Sciences Tulln, Tulln, Austria

**Keywords:** parents with mental illness, children of parents with mental illness, intergenerational transmission of mental illness, risk and protective factors, systems change, parents with substance use disorders, children of parents with substance use disorders

## Introduction

Families living with parental mental and substance use disorders face considerable biopsychosocial and, oftentimes, socioeconomic challenges, with complex pathways to mitigating risk, enhancing resilience in children and youth, and supporting recovery in adults who are parents. This special issue contains the latest knowledge on a) the prevalence of parental psychiatric disorders and children who may be affected, and relationships among risk and protective factors and outcomes for children, youth, and parents across the lifespan, including the perinatal period; b) intervention development, implementation, and testing at the individual practitioner, parent, child, or youth level, as well as recommendations for making change at the national level; and c) innovative measurement and methodological developments, including the protocols of studies currently underway. The special issue comprises 26 papers, representing the contributions of nearly 100 investigators from 15 countries in Europe, Asia, and North America. The research studies embrace diverse designs and methods; and both primary data collection with providers, parents, children, and youth, and secondary analyses of national and registry data sets. Several papers describe innovative research measures and methods, exciting developments in the field. Review and publication of the protocols of current studies serve to enhance research integrity and rigor, and guide next steps in future studies.

## The Prevalence of Parental Mental Illness and Related Characteristics and Conditions

How many individuals in treatment in Adult Mental Health Services (AMHS) have children younger than 18 years of age who may need support or benefit from intervention? So far, data to answer this simple question have been sparse and inconsistent. Ruud et al. have obtained registry data from 23,167 receiving outpatient treatment in Norway at 107 AMHS and found that among them 36% had children under 18 years of age. Referrals to health and social services were made for children of one of three outpatients (with young children), while for a substantial proportion of these children it was unknown whether they had met or unmet needs. This finding suggests that the proportion of patients in treatment who have children who may need support is relevant, and requires appropriate actions.

Also, in Norway, Reedtz and co-investigators assessed a large sample (n = 422) of parents with mental illness receiving treatment and their young children (n = 581), and found that three quarters of them (76.2%; n = 526) were living with the ill parent. One third of the sample (32.5%; n = 170) lived with a single ill parent and with siblings, full-time or part-time. Younger children did not receive any information about their parents' disorders. Overall, this study confirms that a large percent of offspring spend a substantial amount of time with ill parents, and when parents are separated or divorced the contact with the ill parent may become particularly intensive, with an increased exposure to potentially stressful situations.

In their contribution, Moscoso et al. show that, in the family history of adolescents diagnosed with borderline personality disorder, there may be parental suicidal attempts, and the number of suicidal attempts is related to an increased severity of the adolescent's disorder. Children of parents who have attempted suicide, or completed suicide, are a particularly vulnerable population, who should receive targeted preventive interventions. Unfortunately, this rarely happens, even in countries with well-developed mental health services. This finding points to the need for a careful reconfiguration of services to enable effective response to potential users' needs.

An important study on a large sample of over 2,200 twins born in the United Kingdom in 1994–95 and followed up to the age of 18 has been conducted by Riches and collaborators. The authors assessed both the twins' mothers, when the children were ages 10 and 12, and the children themselves at ages 12 and 18. They assessed a variety of potential protective factors in relation to the development of sub-clinical psychotic phenomena among those children of mothers suffering from psychotic disorders. The authors found that good cognitive functioning, living in a more socially cohesive neighborhood and higher levels of perceived social support were independently protective against early psychotic symptoms among children of mothers with psychosis at ages 12 and 18. This underscores the importance of psychosocial factors in buffering the biological risk associated with having a mother with a severe psychotic disorder.

Three papers deal with perinatal issues affecting mother and child emotional life. Aktar et al. reviewed the literature analyzing early exposure to parental psychopathology, during the pregnancy and in the first *post-partum* year, and its association with child psychological functioning beyond the *post-partum* period, up to adulthood years. They conclude that early interventions targeted in particular to mothers showing severe emotional disturbances in the perinatal period may have beneficial effects on the child for periods which extend to adult life. Once again, the importance of early and timely interventions is confirmed, to effectively intervene with mental disorders at their onset, stopping the chain of intergenerational transmission of these pathological conditions.

Risk factors in the perinatal period and their effects on newborns were the focus of two studies. Ichikawa and colleagues have evaluated the association between low levels of prenatal alcohol exposure (PAE) and children's health. They assessed a large sample of children under 18 years old who have siblings (n = 1,600) and their mothers. Children were evaluated with the CBCL, while mothers' PAE was assessed retrospectively. Low PAE was associated with children's anxiety, internalizing problems and overall health problems, pointing to the importance of screening mothers for PAE to adopt timely preventive interventions and increase the odds of healthy child development.

Sheeba B et al. screened 280 pregnant mothers attending an antenatal clinic in Bangalore, India. Over 1/3 of the sample were found to screen positive for prenatal depression, and the risk was much higher for women exposed to domestic violence or to stressful life events, again highlighting the need for the delivery of appropriate screening interventions targeting emotional well-being among pregnant women. Screening and assessment can promote the implementation of timely interventions, with a beneficial double effect on the mother and on the newborn.

This set of papers highlights the role played by a parental mental disorder in the overall functioning of the family system, and once again underscores the need for appropriate mental health prevention, intervention and promotion. Even simple approaches can have an enduring effect contributing to children's outcomes over the life span.

## Intervention Development, Implementation, and Testing

A number of contributions to this special issue represent efforts to develop, implement, and test interventions involving practitioners, as well as parents and children or youth. In Portugal, van Doesum and colleagues targeted professionals from a hospital psychiatric service received training in the Child Talks intervention. Professionals' reports regarding attitudes, knowledge, confidence, and organizational support improved significantly, as measured by the Family-Focused Mental Health Practice Questionnaire prior to training and 10 months later. In Hafting et al.'s paper, the authors integrate findings from a series of studies on the capacity of General Practitioners (GPs) to identify and provide support to the children of ill and substance-abusing parents. Given their finding that both parents and GPs are ambivalent about addressing these issues during consultation visits, the authors make recommendations for GPs to gain knowledge about the family situation.

Norwegian colleagues, Lauritzen et al., describe 5-year follow-up findings from a study in which two interventions were implemented to support healthcare professionals in identifying and providing support for children of patients within AMHS. While some changes in clinical practice were found, the authors conclude that practice change is a very time-consuming process. In the USA, Nicholson and Valentine interviewed key informants as the first step in designing a model of parent-peer supports for parents with psychiatric disorders. While peer supports are burgeoning in the USA in other domains, the authors' findings raise key issues in practice with parents, including the training needs and organizational infrastructure to support both parent-peer specialists as well as parents themselves.

Children and adolescents living with parental mental illness, their families and professionals who work with them were engaged in co-producing an 8-week group intervention, Young SMILES, for 6- to 16-year olds and their parents, as described by Gellatly and collaborators. This leading UK effort lays the groundwork for meeting the needs of a broad range of ages and levels of need, and for rigorous research to evaluate the impact. Reupert and colleagues target young adults aged 18 to 25 years, with a parent with a mental health and/or substance use disorder, in laying the groundwork for the development for an online group intervention in Australia, “mi.spot.” They suggest implementation considerations and directions for future testing. Potijk and colleagues in the Netherlands addressed parents' hesitancy to let children participate in preventive programs through a pilot study in which a psychoeducation program on parenting and mental illness for parents was developed. They make recommendations regarding implementation as well as for future research. Importantly, mothers' identity is placed in the context of a personal recovery paradigm by Australian authors, Hine et al. The authors suggest that services may promote recovery by supporting the enhancement of their self-concept associated with mothering, in addition to attending to other attributes and roles.

Systems-level change is addressed by Isobel et al. in their study of the stories of change provided by an international sample of systems' change experts from 16 countries. While the issues facing children, parents and families may share similarities across countries, the pathways to systems-level change among countries have unique aspects as well as areas of overlap. This paper provides an interesting global perspective. In Finland, the Let's Talk model was implemented in three municipalities, supporting stakeholders and families in working together to enhance outcomes for children. Niemela and colleagues compared data from these municipalities with national data, and demonstrated a significant decrease in referrals to child protections services.

## Research Innovation

This issue also provides insight into cutting-edge research endeavors in the field. *Postpartum* psychiatric disorders such as depression, anxiety, or psychosis occur in about 15% of mothers and negatively affect their caregiving behavior at an early stage of child development. In response to shortcomings of existing measurement tools, Heinisch et al. have developed an adapted method to assess maternal sensitivity based on methods recently approved in attachment research. Initial application of the new measurement tool showed different deficits in mothers' caregiving behavior depending on diagnosis. At the same time, many mothers were well capable of behaving in sensitive manner toward their child despite their illness. The new method is open for use with pending reliability testing.

Children of parents with a mental illness may be challenging to reach for research and interventions. Using a case study analysis, Grove explores social networking sites as a medium to include at-risk youth and their families by removing accessibility barriers. Ethical considerations and limitations of this promising means of engagement are discussed. Riebschleger and colleagues provide further validation of the Knowledge of Mental Illness and Recovery (K-MIR) scale to examined mental health literacy levels and coping outcomes for youth, which they used before and after a school-based mental health literacy program, Youth Education and Support (YES). Their study also shows a positive effect of the intervention on mental health literacy and coping strategies.

Research currently underway provides promise of exciting future findings. Thorup and co-investigators introduce the follow-up of their large registry based cohort study, which will assess 522 children born to parents with schizophrenia, bipolar disorder and controls as they turn 11 years of age. This study will determine whether the children at familial risk reveal delayed developmental courses, but catch up at age 11, or whether the discrepancies between the groups have grown even larger. It will also shed light on aspects of resilience and neurobiological outcomes.

The Village project is described by Christiansen et al., in which they seek to improve child development and wellbeing outcomes for children of parents with a diagnosed mental illness. The project encompasses co-development, implementation, and evaluation of a practice approach to the early identification and collaborative care, through establishing child-focused support networks and using innovative open innovation science (OIS) approaches.

Christiansen and colleagues describe the part I protocol for a randomized controlled trial comparing parental cognitive behavioral therapy/CBT with CBT + Parenting Program for parents with a mental disorder, flanked by four add-on projects that apply behavioral, psychophysiological, and neuro-imaging methods to examine potential moderators and mediators of risk transmission. This large, multicenter study provides opportunity to test the components of the transgenerational transmission of mental disorders. The part II protocol for the study is provided by Stracke and colleagues.

Steardo and co-investigators are conducting a *randomized controlled trial* for pregnant women with high *postpartum* depression scores. Women will be randomized to receive a psychoeducational intervention or a best practice control condition. The authors will not only develop an informative package for pregnant women and promote a screening program for women with *postpartum* depression, it will also identify factors associated with a higher risk of developing perinatal or postnatal depression, and evaluate the effectiveness of the psychoeducational intervention for reducing symptoms during pregnancy.

Finally, Reedtz and an international group of colleagues introduce the creation of a research and intervention model to promote large-scale implementation and evaluation of generic, brief interventions for children of parents with mental disorders. This multi-site international protocol allows for assessment of both minor children aged 6 to 18 years and their parents, and draws from both electronic patient journals as well as primary data collection using standardized measures. Child Talks+, developed in the Netherlands, will be implemented in participating countries.

## Conclusion

The papers in this special issues represent a significant contribution to building the evidence base of effective interventions for family members who are living with or who are parents with mental and substance use disorders, and lay the groundwork for future innovation in research, screening and assessment, intervention development, and testing. The evidence may be applied to effect innovation or change in policy and practice, and suggests the need for widespread knowledge translation and dissemination, highlighting the significant potential to improve public mental health in countries throughout the world.

## Author Contributions

JN, GdG, and BS contributed substantially to the development of this special issue, and to the review and editing of all papers included. JN, GdG, and BS actively contributed to the preparation and review of the editorial manuscript.

## Conflict of Interest

The authors declare that the research was conducted in the absence of any commercial or financial relationships that could be construed as a potential conflict of interest.

